# Facial subcutaneous emphysema after tonsillectomy

**DOI:** 10.1186/1746-160X-10-11

**Published:** 2014-04-11

**Authors:** Argyro Bizaki, Janne Kääriäinen, Teemu Harju, Markus Rautiainen

**Affiliations:** 1 Department of Otolaryngology, University Hospital of Tampere, Tampereen yliopistollinen sairaala, PL 2000, Tampere 33521, Finland

**Keywords:** Subcutaneous emphysema, Tonsillectomy, Complication

## Abstract

**Background:**

Tonsillectomy is a commonly performed and relatively safe surgical procedure. However, it can potentially be associated with several complications. We report a case of facial subcutaneous emphysema that occurred after elective tonsillectomy.

**Case:**

Tonsillectomy was performed on a patient with a history of frequent tonsillitis. After surgery, the patient developed facial subcutaneous emphysema that resolved within a few days without any further complications.

**Conclusion:**

Subcutaneous emphysema is a rare complication of tonsillectomy. Tonsil should be removed along the tonsilar capsule. If its removal causes a deeper than usual mucosal tear up to the level of the muscles, then air might potentially pass through the pharyngeal wall to the parapharyngeal, retropharyngeal and prevertebral spaces.

## Background

Tonsillectomy is a commonly performed surgical procedure in the field of otolaryngology. Although tonsillectomy is a relatively safe surgical procedure, it is nevertheless associated with several complications that include bleeding, infection, lingual edema, injury to the glossopharyngeal nerve, and injury to the carotid artery. In addition, a rare complication of tonsillectomy is cervicofacial subcutaneous emphysema that in most cases resolves spontaneously [1-11]. Nevertheless, subcutaneous emphysema is a potentially life-threatening condition, as it may progress to obstruct the upper airways or spread to the thorax causing pneumomediastinum or pneumothorax and impair cardiorespiratory function [3-5,12,13]. According to the literature, the first case of cervicofacial subcutaneous emphysema was reported in 1936 [14]. We report a case in which facial subcutaneous emphysema developed after elective tonsillectomy.

## Case presentation

### Medical history

A 29-year-old otherwise healthy woman who had frequently suffered from tonsillitis was admitted for tonsillectomy. The preoperative physical examination revealed no other abnormalities. Tonsillectomy was performed under general anaesthesia with orotracheal intubation. The tonsils were removed by electrodissection with bipolar scissors, and haemostasis was achieved using bipolar cautery. There was no remarkable bleeding during the procedure. There were some adhesions between the tonsils and tonsillar beds that were more remarkable on the left side. After the procedure, the patient was monitored in the recovery room and she was discharged a few hour later.

### Clinical presentation

**Figure 1:**
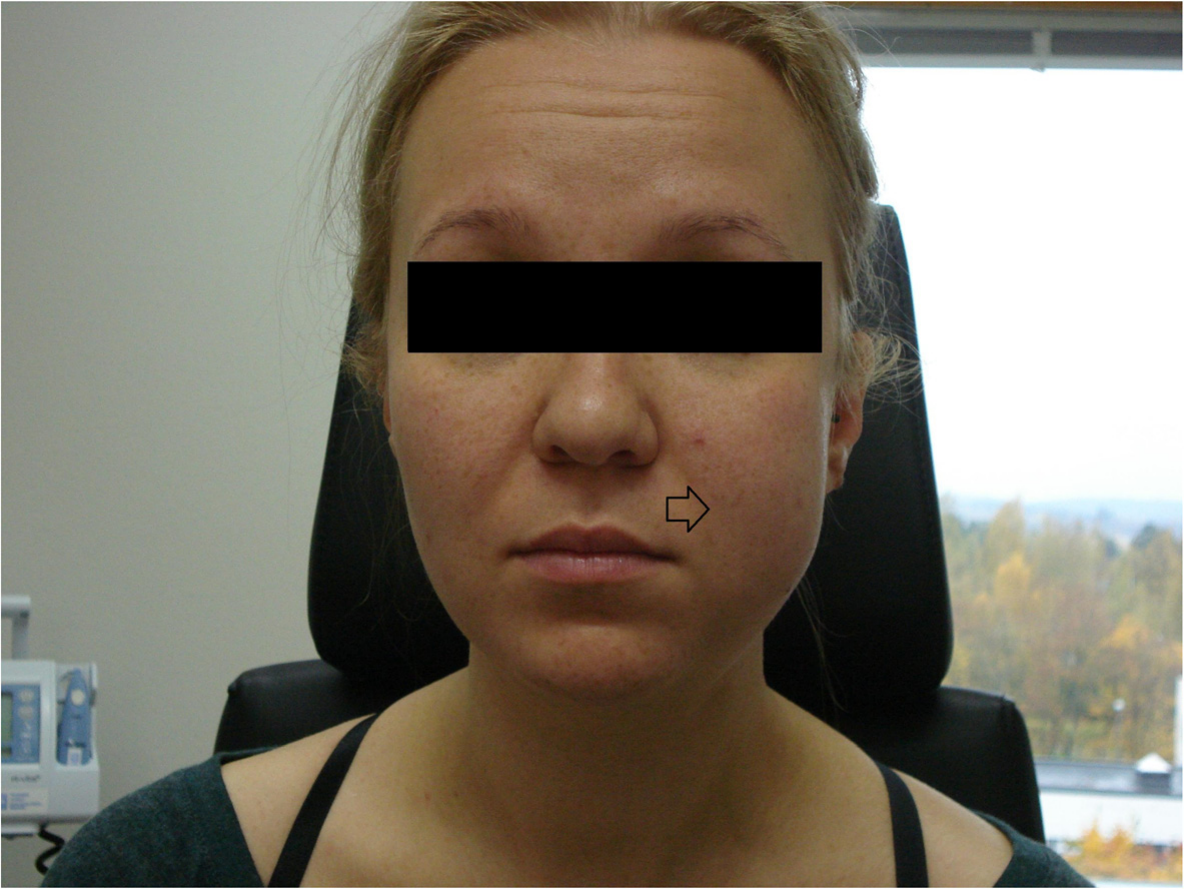
**On the first postoperative day, the left side of the patient’s face was still swollen compared to the right side.** No signs of inflammation were found in the clinical examination.

**Figure 2:**
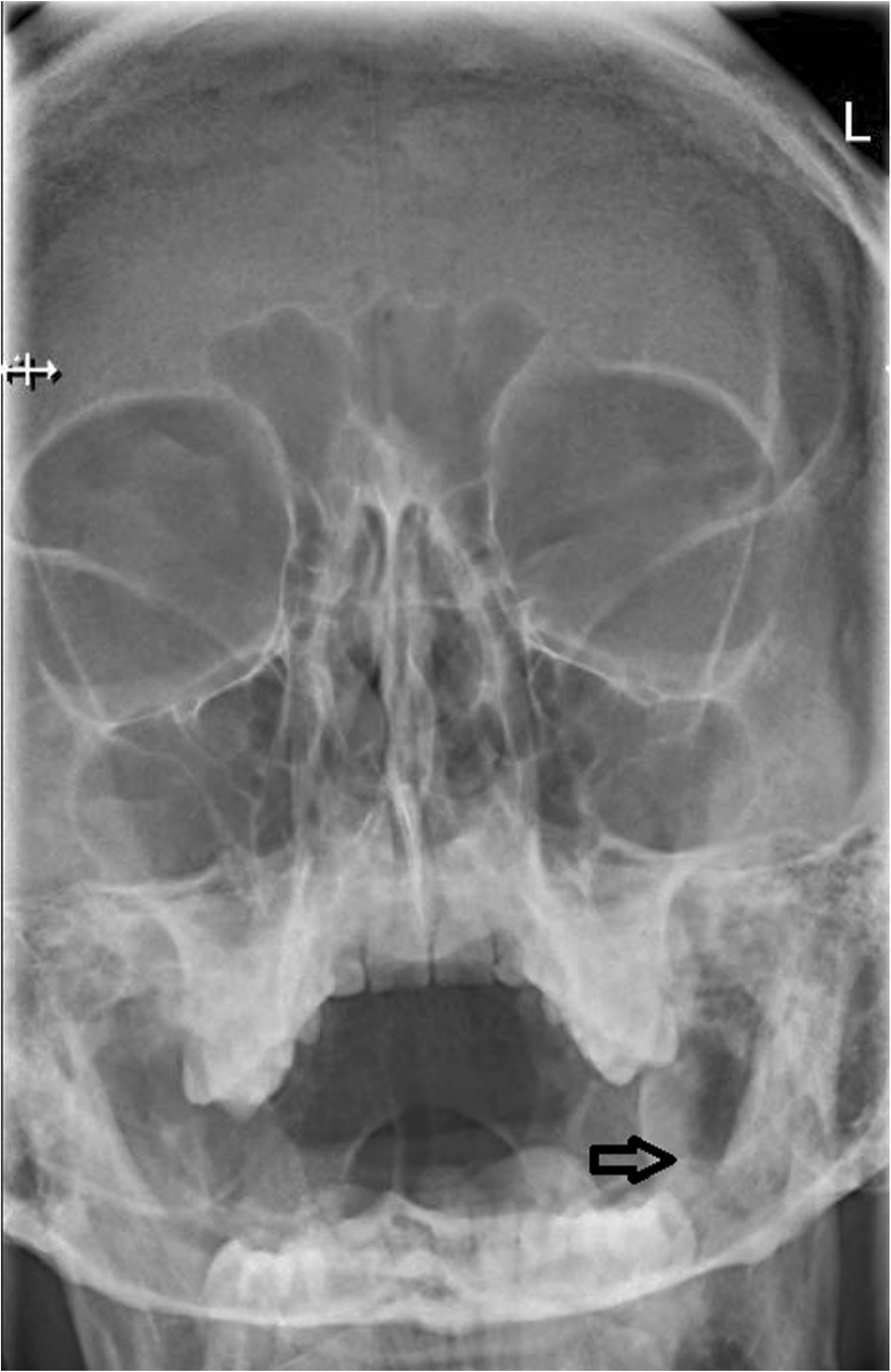
**A plain sinus x-ray was taken at the time of patient’s arrival at the emergency room.** Based on the radiological report, there were no pathological findings. Due to the projection of pneumatized mastoid processes, the plain sinus x-ray has low sensitivity in detecting subcutaneous emphysema in the facial area.

About 14 hours after the procedure, the patient telephoned the emergency department because some swelling had suddenly appeared on the left side of her face, some two hours earlier. She was advised to come to the emergency department immediately. The patient felt some pressure in the facial region and had a sore throat. She had neither a cough nor difficulty in breathing, and she was able to swallow normally. Physical examination by the on-call otolaryngologist revealed left facial swelling and crepitus that extended to the temporomandibular joint region on the same side and also in the upper neck region (see Figure 1). There was neither redness nor signs of cellulitis in the swollen area. Inspection of the tonsillar fossa revealed neither mucosal tear nor other abnormal findings. In indirect laryngoscopy, the larynx and hypopharynx appeared normal. In blood tests, the value of the white blood cells (WBC) was 12.7 × 10^3^/μL and the value of C-reactive protein was 5.1 mg/l. The body temperature was 37.2 degrees Celsius. A chest x-ray was taken the same night and no pathological findings were found. Based on clinical examination, there was no suspicion of abscess or serious infection, and therefore neck computer tomography (CT) imaging was not carried out. Although there was no radiological confirmation the patient was thought to have facial subcutaneous emphysema that developed after the tonsillectomy (see Figure 2).

### Treatment

Treatment was started with a cephalosporin antibiotic (cephalexin 750 mg ×2 per os) and the patient was admitted to the ear and oral diseases ward. The following day, the swelling had extended slightly to the left orbital and frontal areas. The clinical examination of the neck and larynx did not, however, reveal any deterioration. Crepitus was still apparent in the cheek area and also on the mandibular angle. The patient did not have any general symptoms. In blood tests, the WBC was 9.3 × 10^3^/μLand the C-reactive protein was 18.1 mg/l. The antibiotic treatment was continued and the patient was also given oxygen 2 l/min through a nasal cannula for about 24 hours, although she did not have problems with breathing at any point during her stay in hospital.

The next day, the swelling had decreased slightly. The patient was feeling well, and she was therefore discharged. The cephalosporin antibiotic was continued for a week. The patient was also forbidden to smoke.

In the follow-up examination five days after the onset of symptoms, the crepitus and swelling were resolved. In addition, normal post tonsillectomy status was revealed in the pharynx examination without any pathological findings.

## Discussion

Subcutaneous emphysema can be a rare complication of a variety of oral and maxillofacial surgical procedures where mucosal integrity is breached. The mechanism by which subcutaneous emphysema and pneumomediastinum develop after tonsillectomy is not well documented. It has been reported that the development of subcutaneous emphysema after surgery in the oral cavity is caused by injury to the pharyngolaryngeal mucosa [1]. Such injuries to the pharyngolaryngeal mucosa can be caused by surgical techniques and they can also be caused by injury during intubation, excessive positive ventilation, and excessive manual ventilation [2]. In our particular case, on postoperative clinical examination we documented the usual post-operative reaction at the tonsillar fossa with no bubbles seen in the saliva or any usual mucosal tear.

Nevertheless, it was considered that subcutaneous emphysema was caused as sequelae of the tonsillectomy. During surgery, noticeable adhesions between the tonsils and the tonsillar beds were found that made the operation more difficult and might suggest the likelihood of injury to the tonsillar fossa.

It has been reported that deep dissection of the superior pharyngeal constrictor muscle creates a path through the cervicofacial planes to the parapharyngeal, retropharyngeal and prevertebral spaces. Air may go down to the mediastinum through the deep neck spaces and cause pneumomediastinum. In some rare cases, the air that has descended to the mediastinum may then descend further to the abdominal cavity via the diaphragmatic aperture [3-5]. Subcutaneous emphysema is typically associated with crepitus, and subcutaneous air can be detected relatively easily by radiological imaging. It is important to check for pneumomediastinum especially if symptoms such as dyspnea, dysphagia, chest and back pain, cyanosis, and Hamman’s sign (crepitus synchronous with systole) are present [6]. Of the deep neck spaces that are limited above the hyoid bone, the pharyngomaxillary (divided further into lateral pharyngeal, parapharyngeal and peripharyngeal spaces) and the masticator spaces are located closest to the tonsil. Tonsillar fossa is formed anteriorly by the palatoglossus muscle and laterally by the palatopharyngeus and the superior pharyngeal constrictor muscle. In our case, the subcutaneous emphysema was limited to the cheek area extending inferiorly to the submandibular area and posteriorly to the parotid gland area, just under the earlobe. According to the patient’s history, the swelling had first started from the mandible corner just under the earlobe and had extended to the cheek and submandibular subcutaneous tissue.

The literature regarding similar cases was reviewed, and at least a total of 30 similar cases were identified [10]. The indication for the tonsillectomy included frequent episodes of tonsillitis or the previous development of a peritonsillar abscess. Based on the reported cases, the treatment of patients with subcutaneous emphysema and pneumomediastinum involves a regular assessment of the airways and the extent of the emphysema. Any activity that increases upper airway pressure such as coughing, vomiting, straining, or vigorous activity should be avoided. It is appropriate to recommend bed rest and sedation, the restriction of oral intake, and the administration of cough suppressant and stool softener. Broad-spectrum antibiotics may also be prescribed. According to the literature, oxygen therapy has been used in one previously reported case for faster absorption of subcutaneous emphysema [7,8].

In addition, if an examination reveals any macroscopically obvious mucosal tear, the damaged mucosa may be sutured to prevent the secondary entrance of bacteria to the subcutaneous emphysema and the extension of emphysema [6,8]. In most cases, subcutaneous emphysema and pneumomediastinum resolved spontaneously and no deaths have been reported. One case required a tracheotomy [8], and two cases involved a thoracotomy [3,9]. Thus, three cases in total required aggressive treatment. In most of these reported cases, it was suggested that air entered the cervical fascial plane via injury to the superior constrictor muscle during the removal of the palatine tonsils [10]. Even though none of the patients died, subcutaneous emphysema and pneumomediastinum can be potentially fatal complications.

## Conclusion

Therefore, patients undergoing tonsillectomy should be monitored closely. Based on the findings of this case, an extremely meticulous dissection of the tonsil should be performed especially in cases showing severe adhesion between the tonsil and the tonsillar bed. This would most likely decrease the risk of postsurgical bleeding as well as would prevent the development of rare postsurgical complications such as subcutaneous emphysema and pneumosmediastinum.

## Consent

Patient’s consent was received for this case report to be published.

### Level of interest

An article of importance in its field.

## Competing interests

The authors declare that they have no competing interests.

## Authors’ contributions

AB wrote the manuscript, reviewed the literature, treated the patient after the surgical procedure, JK performed the surgical procedure, TH contributed to manuscript’s writing and review of the literature, MR helped to draft the manuscript. All authors read and approved the final manuscript.

## Authors’ information

Bizaki Argyro and Harju Teemu: Specializing medical doctor, Department of Otorhinolaryngology, University Hospital of Tampere, Finland, Kääriäinen Janne: Specialized medical doctor, Department of Otorhinolaryngology, University Hospital of Tampere, Finland, Rautiainen Markus: Professor of Otolaryngology, Department of Otolaryngology, University Hospital of Tampere, Finland.
